# Deep learning assists in acute leukemia detection and cell classification via flow cytometry using the acute leukemia orientation tube

**DOI:** 10.1038/s41598-024-58580-z

**Published:** 2024-04-09

**Authors:** Fu-Ming Cheng, Shih-Chang Lo, Ching-Chan Lin, Wen-Jyi Lo, Shang-Yu Chien, Ting-Hsuan Sun, Kai-Cheng Hsu

**Affiliations:** 1 Division of Hematology and Oncology, Department of Internal Medicine, China Medical University Hospital, China Medical University, Taichung, 404 Taiwan; 2 Artificial Intelligence Center, China Medical University Hospital, China Medical University, Taichung, 404 Taiwan; 3 School of Medicine, China Medical University, Taichung, 404 Taiwan; 4 Neuroscience and Brain Disease Center, China Medical University, Taichung, 404 Taiwan; 5 Department of Neurology, China Medical University Hospital, China Medical University, Taichung, 404 Taiwan

**Keywords:** Computational biology and bioinformatics, Computational models, Machine learning, Haematological cancer

## Abstract

This study aimed to evaluate the sensitivity of AI in screening acute leukemia and its capability to classify either physiological or pathological cells. Utilizing an acute leukemia orientation tube (ALOT), one of the protocols of Euroflow, flow cytometry efficiently identifies various forms of acute leukemia. However, the analysis of flow cytometry can be time-consuming work. This retrospective study included 241 patients who underwent flow cytometry examination using ALOT between 2017 and 2022. The collected flow cytometry data were used to train an artificial intelligence using deep learning. The trained AI demonstrated a 94.6% sensitivity in detecting acute myeloid leukemia (AML) patients and a 98.2% sensitivity for B-lymphoblastic leukemia (B-ALL) patients. The sensitivities of physiological cells were at least 80%, with variable performance for pathological cells. In conclusion, the AI, trained with ResNet-50 and EverFlow, shows promising results in identifying patients with AML and B-ALL, as well as classifying physiological cells.

## Introduction

Flow cytometry is a technique used to analyze the physical and chemical properties of individual cells in a heterogeneous population. This highly sensitive and versatile technology has a broad range of applications, including immunology, and cell biology^1^. Euroflow, established in 2006, is a consortium of European laboratories collaborating to standardize flow cytometry techniques^2^. By harmonizing protocols, reagents, and analysis tools, Euroflow enhances flow cytometry's accuracy, reproducibility, and comparability for research and clinical applications. Acute leukemia orientation tube (ALOT) is one of the most common protocols of Euroflow, designed for screening acute leukemia, including AML, B-ALL, and T-ALL.

However, the analysis of flow cytometry is time-consuming work. Moreover, the bias of interpretation is hard to avoid with gating manually in different physicians or technicians.

In recent years, AI has emerged as a promising approach to enhance flow cytometry analysis^3–6^. Most existing AI relies on traditional machine learning. Deep learning is a machine learning technique that relies on artificial neural networks with multiple layers, enabling the extraction of complex features and patterns from large datasets. The application of deep learning in flow cytometry has the potential to improve the efficiency of analysis and avoid the bias of interpretation.

Here, we present a deep learning application in flow cytometry produced by the acute leukemia orientation tube (ALOT) protocol established by Euroflow.

## Methods

### IRB

The study was approved by the Ethics Review Board of the China Medical University Hospital ethics committee, under the IRB number CMUH111-REC2-137. The requirement for informed consent was waived by the committee. In addition, our research adhered to the principles set forth in the Declaration of Helsinki.

### Protocol of acute leukemia orientation tube

The acute leukemia orientation (ALOT) panel and instrument setting protocol were modified from the EuroFlow Standard Operating Procedure (SOP)^2^. Both peripheral blood and bone marrow samples were obtained from patients in the Department of Hematology and Oncology, China Medical University Hospital. Samples were stained with the following eight antibodies, CyCD3-Pacific Blue (UCHT1, BD Biosciences), CD45- KrO (J.33,Beckman Coulter), CyMPO-FITC (2C7, Cytognos, BD Biosciences), CyCD79a-PE (HM57, Cytognos, BD Biosciences,), CD34-PerCP-Cyanin5.5 (8G12, BD Biosciences), CD19-PECyanin7 (J3-119, Beckman Coulter), CD7-APC(124-1D1, eBioscience), and SmCD3-APCH7(SK7, BD Biosciences). Data were acquired by BD FACSuite software in a BD FACSLyric TM cytometer equipped (both from BD Biosciences) according to the manufacturer’s instructions and EuroFlow SOP. Each acquired data was exported as a Flow Cytometry Standard file (FCS) containing up to 250,000 events. Each event represents a cell with 12 channels of cell information including strength of eight antibodies, forward scatter-A, forward scatter-H, side scatter-A, and side scatter-H.

### Data

This study conducted a retrospective analysis dataset from China Medical University Hospital, spanning the period between 2017 and 2022. We included FCS files produced by ALOT protocol. FCS files without diagnosis are excluded.

### Model training and validation

We split the data into training and testing sets. The training subset contributed 80% of the total data and the testing subset made up the remaining 20% of the data. For cross-validation purposes, the training set was further divided into five folds. Our artificial intelligence training process consists of three phases.

In phase I, we introduced FCS files, which contained up to 250,000 events with 12 channels and the diagnoses of each patient within the training set. We utilized the ResNet-50 model as our training mechanism, aiming to assess see how the proposed AI identifies patients’ diseases based on 12 channels’ data.

In phase II, we segregated the FCS files manually based on cell types, which resulted in distinct FCS files, each containing a single cell type. Every event in new FCS files still had 12 channels as those in phase I. We then inputted these new FCS files and the labels of the cell type. In this phase, we employed the EverFlow model, described in the later section, to train AI’s capability to recognize each cell type.

In phase III, we utilized the AI, previously trained in phase II by EverFlow, to analyze each patient’s cellular composition initially. Subsequently, the composition, either with or without the 12 channels, along with the diagnoses of each patient were introduced into the AI training. ResNet-50 was chosen as the training model of phase III to train AI to identify patients' diseases.

### ResNet-50

ResNet-50 is a deep residual neural network architecture, which is effective for image classification tasks^7^. It is a 50-layer deep neural network that is capable of achieving state-of-the-art performance on a variety of computer vision tasks. ResNet-50 was used in phases I and III of our artificial intelligence training to identify patients' diseases.

### EverFlow

In phase II, we designed a multi-level network architecture called EverFlow to recognize each kind of cell. EverFlow is a versatile and advanced multi-level network architecture specifically designed for analyzing data from FCS measurements. The EverFlow architecture features a sophisticated combination of three Conv1d layers (convolutional layers), three BatchNorm1d layers (batch normalization layers), and two MaxPool1d layers (max-pooling layers), integrated with the activation function ReLU and an Adaptive Average Pooling layer to optimize the computational resources, enhance the precision of the analysis, and adapt to the dynamic nature of flow cytometry data.

**Figure 1:**
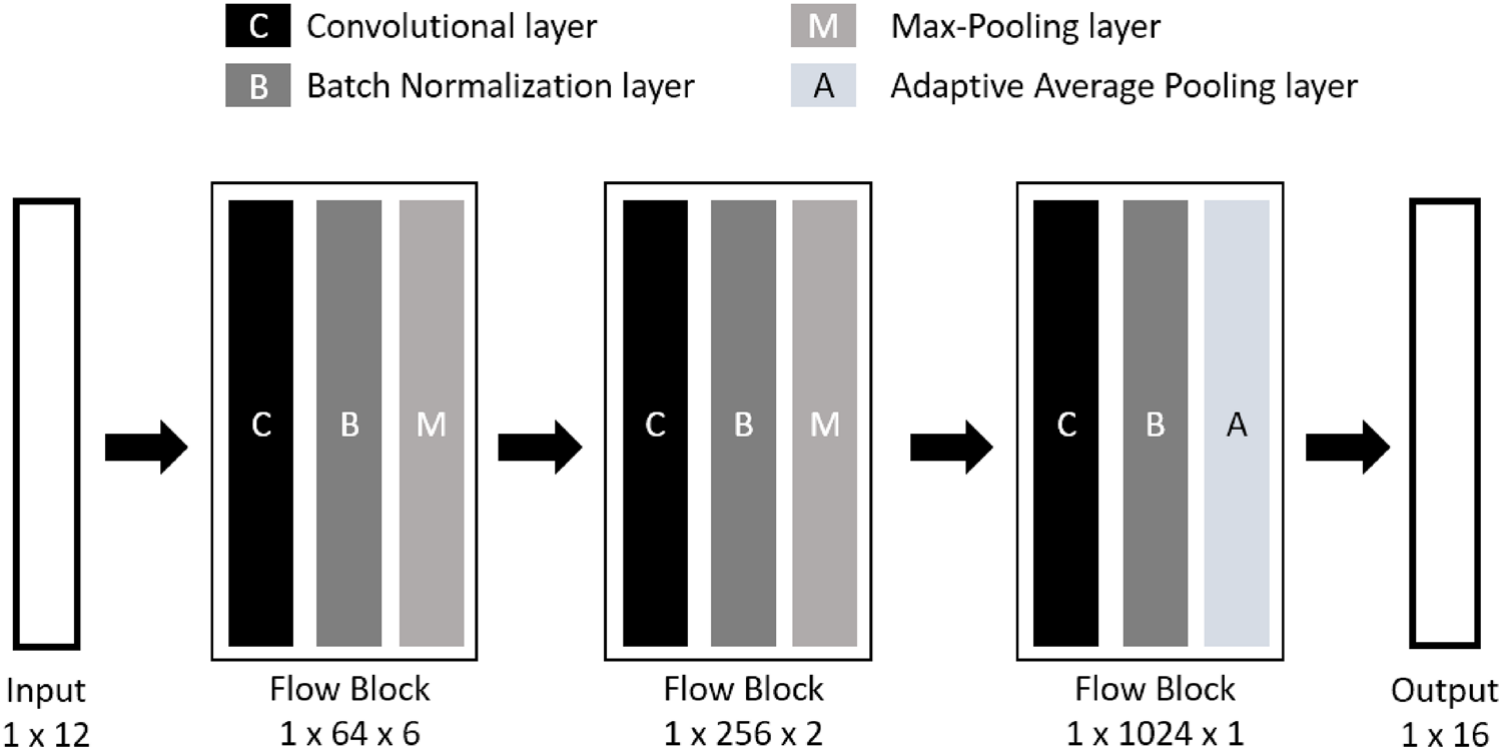
EverFlow progress on a multi-level network architecture which includes three Conv1d layers (convolutional layers), three BatchNorm1d layers (batch normalization layers), and two MaxPool1d layers (max-pooling layers). Additionally, we utilize the activation function ReLU and an Adaptive Average Pooling layer. This combination optimizes the computational resources required by EverFlow, enhances the analysis efficacy, and caters to the growing demands of modern networks. For training, we leverage the Ranger optimizer^8^ with a learning rate of 5E−3. The training process typically converges within 75 epochs.

EverFlow also introduces a architectural component, the Flow Block, which combines Conv1d, BatchNorm1d, MaxPool1d, and ReLU layers. The Flow Block significantly improves the network's ability to discern complex features and patterns within the FCS data, leading to improve overall efficiency and performance of the model for flow cytometry analysis tasks (Fig. 1). By concatenating multiple Flow Blocks, EverFlow efficiently encapsulates hierarchical data representations, delivering exceptional outcomes across various flow cytometry data analysis applications.

### K-fold cross-validation

We used K-fold cross-validation to evaluate the performance of our artificial intelligence model in all three phases. The K we used in this study is five. K-fold cross-validation is a technique used in machine learning to evaluate the performance of a model on a limited dataset. The dataset is split into k equally sized folds, and the model is trained and tested k times. Each fold is used once as the test set and the remaining folds are used as the training set. By using k-fold cross-validation, it is possible to obtain a more accurate estimate of model performance and avoid overfitting the training data ^9^.

## Results

### Data

This study conducted a retrospective analysis that included a total of 241 patient datasets from China Medical University Hospital, spanning the period between 2017 and 2022. The dataset was divided into five distinct groups for analysis purposes. These groups consisted of 41 patients diagnosed with AML, 43 patients with B-ALL, 60 patients with complex conditions requiring further analysis (potentially indicating myelodysplastic syndrome or being insignificant, 64 patients with normal findings in flow cytometry, and 34 patients with other diseases, including T-ALL, B-cell lymphoma, T-cell lymphoma, myeloma, and hemophagocytic lymphohistiocytosis. All three phases of our AI training utilized the above data, and the results are presented separately as follows:

### Phase I

In this phase, a total of 185 patients’ datasets were included in both the training sets and validation sets using the method of five-fold cross-validation. An additional 56 patients’ dataset was employed for testing. Each patient’s data consisted of 12 channels listed in the protocol of acute leukemia orientation. The accuracy of recognizing AML was 91.1% (51/56), demonstrating a sensitivity of 80.0% (8/10), and a specificity of 93.5% (43/46). Furthermore, the accuracy of recognizing B-ALL stood at 94.6% (53/56), demonstrating a sensitivity of 90.91% (10/11) and a specificity of 95.6% (43/45). More detailed information is displayed in Table 1. Both sensitivity and specificity of AML and BALL are promising.

**Table 1:** The testing results of Phase I; abbreviations: AML = acute myeloid leukemia, B-ALL = B-acute lymphoblastic leukemia.

Disease	F1	Accuracy	Sensitivity	Specificity
Normal	60.0%	78.6% (46/56)	69.2% (9/13)	81.4% (35/43)
AML	76.2%	91.1% (51/56)	80.0% (8/10)	93.5% (43/46)
B-ALL	87.0%	94.6% (53/56)	90.91% (10/11)	95.6% (43/45)
Complex	44.4%	73.2% (41/56)	42.9% (6/14)	83.3% (35/42)
Other disease	54.6%	91.1% (51/56)	37.5% (3/8)	100% (48/48)

### Phase II

**Figure 2:**
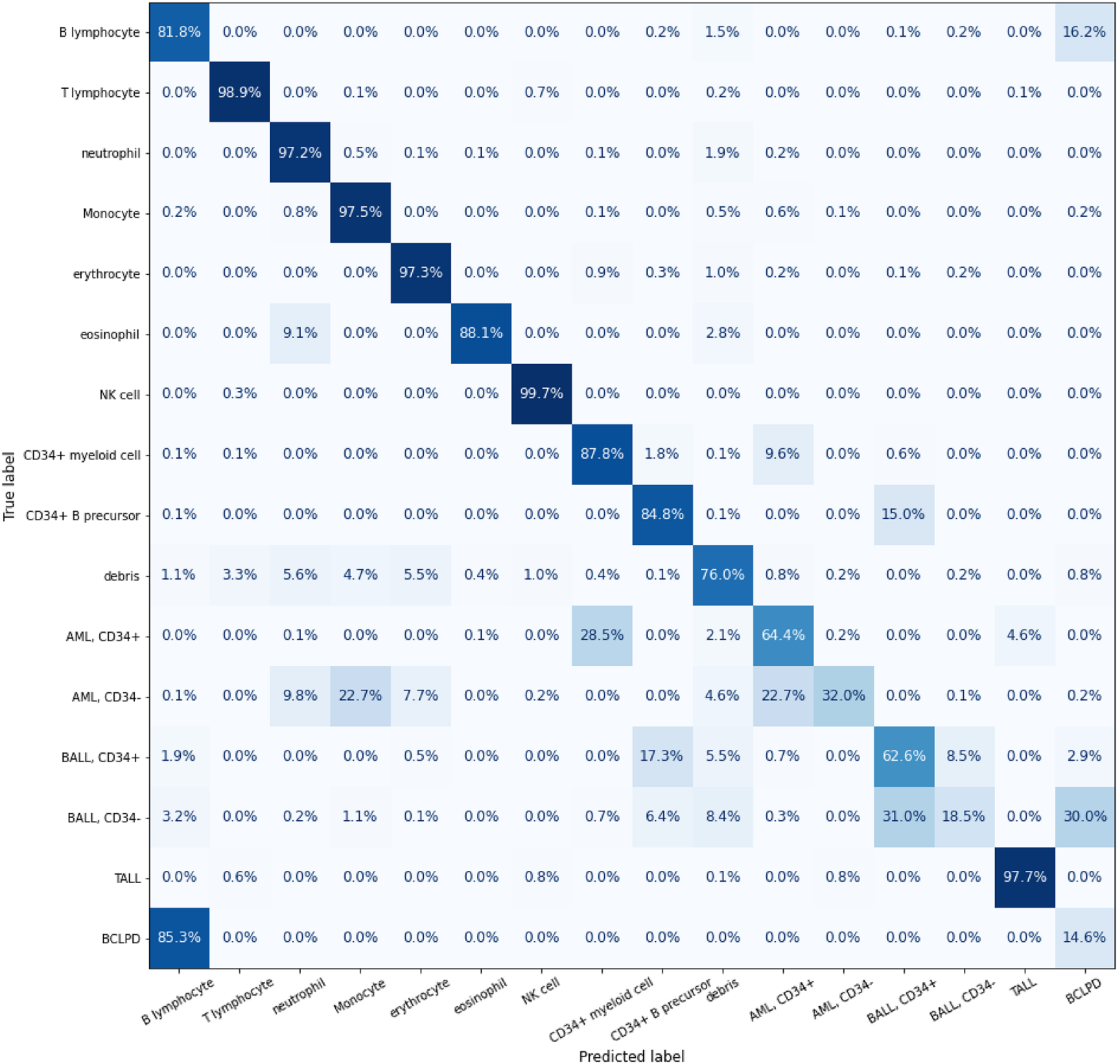
The rates of cells properly identified by AI; Abbreviations: AML = acute myeloid leukemia, B-ALL = B-acute lymphoblastic leukemia, BCLPD = B-cell lymphoproliferative disorder, NK = nature killer, T-ALL = T-lymphoblastic leukemia.

**Figure 3:**
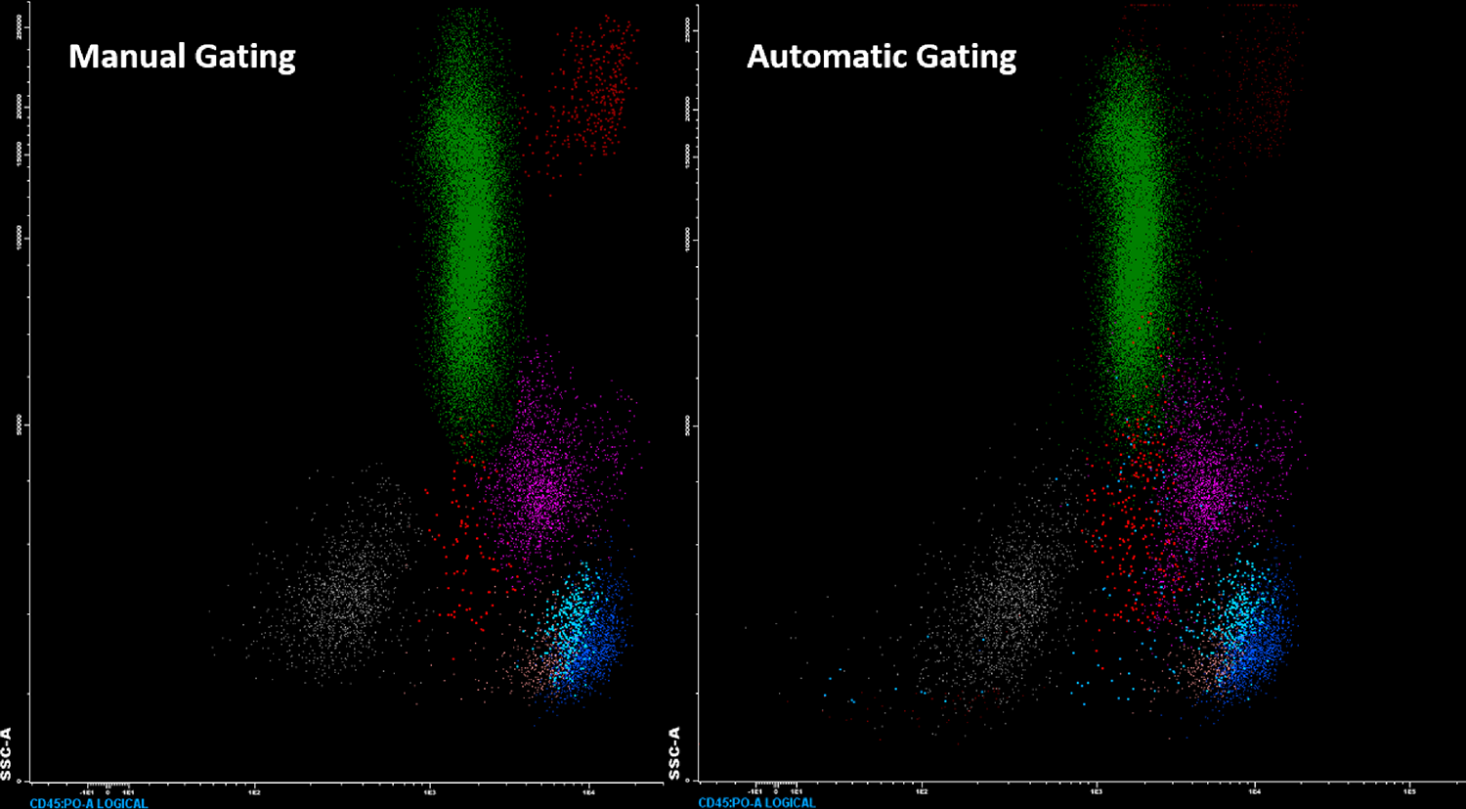
With EverFlow, trained in Phase II, we can efficiently classify cells type. The left side of this figure shows an outcome from manually gating, with dots of different color representing various cell types. The right side of this figure presents an outcome generated by Everflow. Two outcomes are highly similar to each other.

We have identified FCS files corresponding to a diverse patient pool: 10 patients diagnosed with AML with CD34-positive, 10 patients with AML with CD34-negative, 10 patients with B-ALL with CD34-positive, 10 patients with B-ALL with CD34-negative, 10 patients with B-cell lymphoma, 10 patients with normal findings and ten patients with T-lymphoblastic leukemia. We conduct a manual gating procedure on these files, subdividing them according to cell type, which in turn led to the creation of distinct FCS files, each embodying a singular type of cell. These new FCS files were included as our training and testing data. (Table 2.) EverFlow was selected as the training model in this phase. In the testing data, the AI identified normal cells with an accuracy of over 80% for most cell types (Fig. 2). To identify the pathologic cells, the efficacy is various with different types of cells. Both AML and B-ALL with CD34-positive were well recognized by AI in 64.4% and 62.6%. The properly identifying rate in AML with CD34-negative dropped to 32.0% and the rate in B-ALL CD34-negative is only 18.5%. Only 14.6% of cells of B-cell lymphoma are properly identified by the AI (Fig. 3).

**Table 2:** The number of each cell type to train AI and to test AI; Abbreviations: lym = lymphocyte, neu = neutrophil, mono = monocyte, ery = erythrocyte, NK = Nature killer, CD34+ M = CD34+ myeloid cell, CD34+ B = CD34+ B-precursor, AML = acute myeloid leukemia, B-ALL = B-acute lymphoblastic leukemia, T-ALL = T-lymphoblastic leukemia, BCLPD = B-cell chronic lymphoproliferative disorder.

Cell type	B lym	T lym	Neu	Mono	Ery	Eos	NK	CD34 + M
Number of Training	21,659	154,384	1,239,472	110,491	168,531	19,543	25,784	10,049
Number of Testing	12,370	51,928	388,069	22,001	26,206	5766	4328	1268
Testing/training	36.6%	25.2%	23.8%	16.6%	13.5%	22.8%	14.3%	11.2%

Cell type	CD34+ B	debris	AML, CD34+	AML, CD34−	B-ALL, CD34+	B-ALL, CD34-	T-ALL	BCLPD
Number of Training	3971	729,473	1,131,283	963,743	1,123,584	1,145,780	968,724	486,071
Number of Testing	906	93,099	263,669	506,688	270,430	299,910	206,789	71,828
Testing/training	18.6%	11.3%	18.9%	34.5%	19.4%	20.8%	17.6%	12.9%

### Phase III

In this phase, similar to phase I, a total of 185 patients’ datasets were included in both the training sets and validation sets using the method of five-fold cross-validation. An additional 56 patients’ dataset was employed for testing. The inputted data consisted of cell compositions of each patient with or without 12 channels.

Without 12 channels, the accuracy of recognizing AML was 89.2% (50/56), demonstrating a sensitivity of 90.0% (9/10), and a specificity of 89.1% (44/46). The accuracy of recognizing B-ALL was 98.2% (55/56), demonstrating a sensitivity of 90.91% (10/11) and a specificity of 100% (45/45). With 12 channels, the accuracy of recognizing AML was 94.6% (53/56), demonstrating a sensitivity of 90.0% (9/10), and a specificity of 95.7% (44/46). The accuracy of recognizing B-ALL stood at 98.2% (55/56), demonstrating a sensitivity of 90.9% (10/11) and a specificity of 100% (45/45) Tables 3. and 4. Combining outcomes from phase I and Phase III, our AI effectively identified patients with AML or B-ALL using data from either the 12 channels of FCS files, cell composition, or a combination of both. The performances are nearly identical; however, the AI appears to perform optimally when provided with information from both 12 channels and cell composition.

**Table 3:** The testing results of Phase III with cell label only.

Disease	F1	Accuracy	Sensitivity	Specificity
Normal	64.5%	80.4% (45/56)	76.9% (10/13)	81.4% (35/43)
AML	75.0%	89.3% (50/56)	90.0% (9/10)	89.1% (41/46)
B-ALL	95.2%	98.2% (55/56)	90.91% (10/11)	100% (45/45)
Complex	50.0%	82.1% (46/56)	35.7% (5/14)	97.6% (41/42)
Other disease	75.0%	92.9% (52/56)	75% (6/8)	95.8% (46/48)

**Table 4:** The testing results of Phase III with cell labels and data of 12 channels.

Disease	F1	Accuracy	Sensitivity	Specificity
Normal	60.0%	78.6% (44/56)	69.2% (9/13)	81.4% (35/43)
AML	85.7%	94.6% (53/56)	90.0% (9/10)	95.7% (44/46)
B-ALL	95.2%	98.2% (55/56)	90.9% (10/11)	100.0% (45/45)
Complex	41.7%	75.0% (42/56)	35.7% (5/14)	88.1% (37/42)
Other disease	75.0%	92.9% (52/56)	75.0% (6/8)	95.8% (46/48)

## Discussion

ResNet-50 was chosen for the training in Phase I and Phase III of our project. This decision was made because it showed excellent performance in Phase I. In this phase, we tried out several models, which included CNN, SEResNet-50, SEResNext50, ResNext50_32 × 4d, and Efficientnet-B4. Even though all these models did well, ResNet-50 was the best in terms of speed and accuracy. Therefore, we chose ResNet-50 for Phase III as well.

However, for Phase II, we found that ResNet-50 wasn't the right fit. In this phase, we used AI to identify different types of cells, which was quite different from Phase I and Phase III where AI was used to spot diseases. Because of this, using ResNet-50 for training AI in Phase II led to overfitting, even though it worked well in Phase I and Phase III. So, we came up with EverFlow, a simpler model that we created specifically to analyze FCSs in Phase II.

Our AI effectively identified patients with AML or B-ALL. Despite its impressive performance, the AI still faced difficulties in correctly identifying one patient with AML and one with B-ALL. The AML patient, whom the AI failed to identify, had AML cells with CD34-negative. In the context of ALOT, these CD34-negative AML cells may not have the specific marker required for accurate identification. This could lead to confusion and inaccuracies, even when categorizing the cells manually. The AI classified this patient as a complex case requiring further analysis. Strictly, all patients with AML are successfully identified ultimately. In a similar vein, the AI failed to accurately identify a patient with B-ALL whose cells were also CD34-negative. These cells can easily be misclassified as B-cell lymphoma in a clinical setting, which is precisely the mistake made by the AI. In conclusion, AI may have limitations in identifying pathological cells that are CD34-negative, a task that can be challenging even when performed manually.

In phase II, our AI only identified 64.4% AML cells and 62.6% B-ALL cells that were positive for the CD34 marker. Despite these percentages, the AI, which was trained on cell composition, was still able to identify all patients with either CD34-positive AML or CD34-positive B-ALL. Moreover, some cells like basophils could not be identified in ALOT tube may be misrecognized as other cells by AI. This suggested that the AI only needed to identify a subset of pathological cells to make a correct diagnosis. Even with a lower sensitivity of 32.0% for CD34-negative AML cells and 18.5% for CD34-negative B-ALL, the AI maintained a robust performance in patient identification. This performance was, at a minimum, comparable to that of a human, as we discussed in the previous section.

Myelodysplasia syndrome also referred to as preleukemia, another disease that clinicians aim to detect using flow cytometry. However, ALOT was designed primarily for screening acute leukemia and the sensitivity for detecting myelodysplasia syndrome remained uncertain. Another protocol established from Euroflow, named acute myeloid leukemia/myelodysplasia syndrome tube (AML/MDS) which utilizes a far more complex set of antibodies, is created for the diagnosis of myelodysplasia syndrome. In our study, we classified patients showing possible signs of myelodysplasia syndrome as complex cases who were recommended for additional tests with AML/MDS tubes. Within the context of ALOT, the AI struggled to screen these patients with a top sensitivity of merely 42.9%. The current priority of our group is finding effective ways to train AI to identify these patients.

The analysis of flow cytometry typically involves identifying cells based on their expression of specific markers, which is then compared to the expressions in other cells within the same patient. In phase II of our AI training, we amalgamated identical cell types from various patients. This approach, however, resulted in the loss of certain information within individual patients. The consequences of this information loss on the AI’s ability to correctly identify cells remain uncertain. A more thorough investigation is necessary to determine the potential impact of this issue.

In addition to AML and B-ALL, ALOT is also designed for detecting T-ALL. However, patients with T-ALL are relatively less common compared to patients with AML and patients with B-ALL, and in this study, we were only able to include 11 patients with T-ALL. The limited number of T-ALL cases presents a challenge for adequate AI training or further testing to accurately identify patients with T-ALL.

Despite this limitation, in phase II, where the AI is trained to distinguish pathological cells from normal cells, the AI was able to identify T-ALL cells with a sensitivity of 97.7%. This performance was much better than that of AML, CD34-positive, and B-ALL, CD34-positive which achieved sensitivities of 64.4% and 62.6% respectively. This result is logical, given that T-ALL cells, which are CD45-negative, can be more readily differentiated from normal T cells that are invariably CD45-positive. In contrast, pathological AML, CD34-negative, and B-ALL, CD34-negative cells can be easily mistaken for physiological cells, a factor we have noted previously. Therefore, given the effectiveness of AI in recognizing T-ALL cells, we anticipate that AI could also be successful in identifying patients with T-ALL.

Although the sample size employed in this study is relatively limited, the performance of the trained AI in detecting disease and classifying cell types remains promising. The results indicate the benefit of including a larger dataset in future and investing in other protocols of Euroflow.

Comparing to manual gating which take five to ten minutes to analysis an ALOT FCS file, it only takes less than one minute with AI assistance. It saves time efficiently.

There are three features that distinguish our AI from previous studies. First, our training is based on deep learning, while most previous studies relied on traditional machine learning technique. Second, our training focuses on analyzing Euroflow, which is highly standardized and reproducible. Lastly, our AI is trained specifically to detect acute leukemia, while other AIs are trained to identify a variety of diseases.”

## Conclusion

The AI, trained with ResNet-50 and EverFlow, shows promising results in identifying patients with AML and B-ALL, as well as classifying physiological cells. Nonetheless, there remain unmet needs that necessitate further investigation.

### Supplementary Information

Supplementary Information.

## Data Availability

All data generated in this study are included in this published article and its supplementary information files. The data supporting the findings of this study are available upon request. For access, please contact the corresponding author, Kai-Cheng Hsu.
